# The safety and tolerability profile of bilastine for chronic urticaria in children

**DOI:** 10.1186/s13601-019-0294-3

**Published:** 2019-10-23

**Authors:** Nikolaos G. Papadopoulos, Torsten Zuberbier

**Affiliations:** 1 Allergy Department, 2nd Pediatric Clinic, University of Athens, Athens, Greece; 2 Department of Dermatology, Venerology and Allergy, Charité-Universitätsmedizin Berlin, Corporate Member of Freie Universität Berlin, Humboldt-Uniersität zu Berlin, Berlin Institute of Health, Charitéplatz 1, 10117 Berlin, Germany

**Keywords:** Chronic urticaria, Second generation-antihistamine, Bilastine, Children

## Abstract

**Background:**

Urticaria is a condition defined by the development of wheals, angioedema or both. It is classified based on its duration as acute (≤ 6 weeks) or chronic (> 6 weeks). Chronic urticaria is less frequent than acute one in children, but it represents a debilitating condition, always needing treatment. Symptoms affect child’s daily activities and disturb sleeping patterns, causing emotional distress and negatively influencing learning and cognition. Therefore, the management of chronic urticaria must point to a complete control of symptoms, taking into account tolerability and the patient quality of life.

**Review of literature:**

The recently revised version of EAACI/GA^2^LEN/EDF/WAO guideline on the management of urticaria, in addition to recommending the use of second-generation H_1_ antihistamines as the treatment of choice, gives particular attention to their use in the paediatric population. Bilastine has been studied in children; at the dose of 10 mg/once daily, it is licenced for the symptomatic relief of urticaria in children ≥ 6 to 11 years, in the European Union, in appropriate formulation, as oral solution or orodispersible tablet.

**Conclusions:**

In line with the recent guideline recommendation for the use of second generation H_1_ antihistamines in children we have reviewed the safety and tolerability profile of bilastine in children with chronic urticaria.

## Background

Urticaria is a common condition defined by the development of pruritic wheals, angioedema or both [1]. A wheal is characterised by a central swelling of variable size, almost delimited by reflex erythema, itching and a transient nature, with the skin returning normal within 30 min to 24 h. Angioedema, for its part, exhibits a sudden, pronounced erythematous or skin coloured swelling of the lower dermis and subcutis or mucous membranes, sometimes pain and a slower resolution [1].

At the beginning of 2018, an updated version of the EAACI/GA^2^LEN/EDF/WAO (European Academy of Allergology and Clinical Immunology, Global Asthma and Allergy European network, European Dermatology Forum, World Allergy Organization) urticaria guideline was published, providing new inputs regarding both the diagnosis and the treatment of patients with urticaria. This guideline, in the perspective of diagnosis, recommends that urticaria is classified based on its duration as acute (≤ 6 weeks) or chronic (> 6 weeks) [1]. In addition, chronic urticaria is classified as spontaneous (CSU) or inducible (CIndU). CSU is characterized by spontaneous symptoms that are not elicited by apparent factors, and CIndU, on the other hand, requires specific triggers for the urticarial symptoms to occur, such as sunlight, pressure, friction, or exposure to heat or cold [1]. Chronic urticaria is not rare, with a prevalence of at least 1% in the general population and CSU is two to three times more common than CIndU; moreover, patients may have more than one type of chronic urticaria [2, 3]. Urticaria can occur in all age groups, including infants and young children [1] and has similar epidemiological characteristics across different locations [4]. Tang et al. reported that among 411 pediatric patients that visited a dermatological department in China, 314 (76.4%) had acute urticaria and that infection was the main trigger of acute urticaria in children (41%, 16/39). The accompanying symptoms of acute urticaria in children mainly included abdominal pain and diarrhoea (44%, 17/39) [5]. Chronic urticaria is less frequent than acute in children, but it still represents a debilitating condition, always needing treatment. CU in children has a point prevalence between 0.1% and 0.3% and is diagnosed as CSU in 80% of cases. In children, prospective studies suggested that autoimmune CSU affects about half of pediatric CU cases in Europe. Resolution rate in children with CU was found to be lower than in adults (10.3% per year). The presence of certain biomarkers (CD63 level > 1.8% and basophil count) may help to predict the likelihood of resolution [6].

In the management of urticaria, the treatment must aim at complete control of symptoms, taking into account the safety and the patient’s quality of life, as the main goals. Treatment of CU in children should be continuous [1]. Specifically, the use of second-generation antihistamines as a first line therapy is recommended, due to the good safety profile, minimal cognitive and anti-muscarinic side effects, and a long duration of action [1].

Many clinicians still use first-generation, sedating, H_1_-antihistamines as their first choice in the treatment of children because they assume that the safety profile of these drugs is better known. In addition, recommendation about age for the first-generation H_1_-antihistamines is sometimes less clear as these drugs were licensed at a time when the code of good clinical practice was less stringent [1]. A strong recommendation was made by the panel of EAACI/GA^2^LEN/EDF/WAO urticaria guideline, on the basis of current literature, to discourage the use of first-generation antihistamines in infants and children encouraging the usage of the newer non-sedating antihistamines [1].

As understanding the pharmacological characteristics of individual drugs is essential for effective and safer use of antihistamines in clinical practice, this review summarizes the characteristics of bilastine as the most novel 2nd generation antihistamine and describes its use in children with urticaria, as newly recommended by the current guideline. Table 1 compares the safety profile of bilastine with profiles of some representative second generation H_1_ antihistamines (cetirizine, desloratadine, fexofenadine, levocetirizine, loratadine, and rupatadine); first generation drugs are not included in the table, as few data is available and clinical use is discouraged by current guidelines (Table 1) [1].

**Table 1:** Safety profile of representative second generation H_1_ antihistamines indicated for urticaria in children. Modified from 15

	Bilastine	Cetirizine	Desloratadine	Fexofenadine	Levocetirizine	Loratadine	Rupatadine
Properties							
Paediatric indication	Yes	Yes	Yes	Yes	Yes	Yes	Yes
T_1/2_ (h)	14.5, any age	10, in adults 6.1–7.1, in children over 4 years 5.5, in children under 4 years	27, any age	11–15, any age	7.9, in adults In children 6–11 years 24% shorter than in adults	8.4, any age	5.9 in adults 15.9, in children 2–5 years 12.3, in children 6–11 years
Dosage adjustment in impaired kidney function	No	In moderate to severe	In severe impairment	No	In moderate to severe	No	Not recommended in renal impairment
Dosage adjustment in impaired hepatic function	No	If concomitant renal dysfunction	Not mentioned	No	If concomitant renal dysfunction	In severe disease	Not recommended in hepatic impairment
Interaction with food	Yes, give on empty stomach^a^	No	No	Not mentioned	No	No	With grapefruit
Clinically relevant drug interactions	No	No	No	Yes, antacids	No available data	Potential (with inhibitors of CYP3A4 and CYP2D6)	Yes, with CYP3A4 inhibitors
Lack of sedative potential	Yes (caution, drowsiness)	Yes (in adult, check drug response when intending to drive)	Yes (caution, drowsiness)	Yes (impairment is unlikely)	Yes (in adult, check drug response when intending to drive)	Yes (caution, drowsiness)	Yes (caution, drowsiness)
Contraindications (except hypersensitivity)	None	Severe renal impairment	None	None	Severe renal impairment	None	None

^a^Pharmacokinetic interaction of bilastine with food does not imply a significant reduction of its peripheral antihistaminic efficacy [43]

The pediatric indication is also shown. Because of the diversities in regulation between countries worldwide, the youngest age, for which antihistamines are registered according to local resolutions, differs and it is not reported in the table.

## Main text

### Pharmacological basis for the use of antihistamines

Degranulation of active mast cells is the pathophysiological basis of wheals and angioedema, [3, 7]. It leads to the release of histamine and other inflammatory mediators such as platelet-activating factor and cytokines, and results in sensory nerve activation, vasodilatation, and plasma extravasation as well as cell recruitment to urticarial lesions [1]. Many symptoms of urticaria are mediated primarily by the actions of histamine on H_1_-receptors located on endothelial cells (the wheal), on sensory nerves (neurogenic flare and pruritus), in central nervous system cells, smooth muscle cells (blood vessels and respiratory system), chondrocytes, hepatocytes, dendrocytes, monocytes, neutrophils, and lymphocytes [8]. Continuous use of H_1_-antihistamines in chronic urticaria is supported not only by the results of clinical trials but also by the mechanism of action of these medications, that are inverse agonists with preferential affinity for the inactive state of the histamine H_1_-receptor and stabilize it in this conformation, shifting the equilibrium towards the inactive state [1, 9, 10]. Second-generation H_1_ antihistamines are the first line treatment of choice, because of their favourable tolerability profile and the long duration of action. Patients must be instructed to take the drug on a daily basis and not on demand [1].

### Pharmacology, efficacy and safety of bilastine

#### Pharmacological profile

**Fig. 1:**
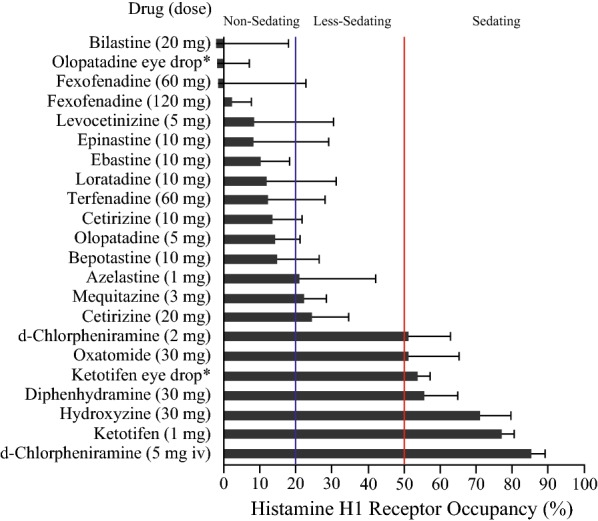
Brain histamine H_1_ receptor occupancies of various antihistamines and classification for sedating actions. The occupancy data are represented as the mean ± SD of measurements in positron emission tomography after oral single-dose, eye drop (*), or intravenous (i.v.) administration of the drugs; the data were obtained by more than one research group. When H_1_ receptor occupancy was 20% or lower, the drug could be classified as “non-sedating” (reproduced with permission from 19)

Bilastine was demonstrated in vitro to have marked selectivity/high affinity for histamine H_1_ receptors, and to have a long residence time at the histamine H_1_ receptor, that may explain the prolonged duration of action [11, 12]. Bilastine is rapidly absorbed after oral administration. [13]. It has a low potential for metabolic drug–drug interaction as it does not interact significantly with the CYP enzyme system in vitro, and it does not undergo significant metabolism in humans [14, 15]. In the wheal and flare test on healthy volunteers, bilastine onset of action occurred within 1 h and reduction of itching sensation was better than that of desloratadine (p < 0.05) and for rupatadine (p < 0.01) [16]. No dosage adjustments are needed in patients with mild, moderate, or severe renal impairment, with hepatic impairment, and in elderly subjects, overall endorsing a good tolerability profile [17, 18]. Bilastine has a high affinity for the P-gP efflux pump, and this effect restricts transit across the blood–brain barrier and limits the potential for sedation [14, 15]. PET (positron emission tomography) showed that bilastine has a brain H_1_ receptor occupancy (H_1_RO) near to 0% and can thus be considered as a “non-brain-penetrating antihistamine” [19, 20]. Brain histamine H_1_ receptor occupancies of various antihistamines are shown in Fig. 1. Bilastine has a potential for negligible central nervous system activity.

### Data from adult studies

#### Efficacy in chronic urticaria

Bilastine efficacy in the treatment of urticaria was demonstrated by a randomized clinical trial in adult patients [21]. Bilastine and levocetirizine were both significantly more effective than placebo regarding reduction in mean total symptoms score, TSS, number of wheals and the maximum wheal size [p < 0.001, days 2–28], and Dermatology Life Quality Index (DLQI) score [bilastine − 9.45 ± 6.98 (p < 0.001), levoce-tirizine − 8.94 ± 6.53 (p < 0.001), and placebo − 5.93 ± 7.67]. In addition, urticaria-associated discomfort (p < 0.001 for change from day 0 to day 28, and p < 0.001 for bilastine/levocetirizine vs placebo) and sleep disturbance (p < 0.001 for bilastine/levocetirizine vs placebo, using Chi square test) were significantly reduced after bilastine or levocetirizine treatment compared to placebo [21]. The efficacy of bilastine was also evaluated vs placebo, and maintained up to 52 weeks in an open-label study in Japanese patients with chronic urticaria [22, 23].

#### Safety and tolerability

For bilastine, a favourable safety profile was observed in clinical trials and in real-life studies both in adults and in children. First, absence of sedation was noticed, as expected for a drug with a H_1_RO near to 0% and considered a “non-brain-penetrating antihistamine” [15, 19, 20].

A review of safety data from well-designed clinical trials published before 2011, included more than 3000 treated patients or volunteers, and concluded that bilastine met the requirements for long duration, effective, and safe therapy [11]. In addition, Yagami et al. assessed the long-term safety of bilastine 20 mg daily for up to 52 weeks in patients with urticaria [23].

As CNS effects are the main tolerability issue for antihistamines, further studies investigated some specific conditions and activities that may be encountered in the real-life setting or may be important for professional or academic reasons and may be related to the H_1_-histamine central receptors. These studies found that bilastine did not interfere with performance in adults in many different activities (such as driving, concomitant administration with alcohol, hypobaric hypoxic condition) [19, 24–26]. These CNS profile in adults suggests that bilastine could be a drug suitable also for children who need that attention was not impaired by therapy. Moreover, in clinical trials, bilastine was not associated with any clinically relevant QTc interval prolongation [11, 27].

### Data from paediatric studies

These reassuring safety data on adult subjects prompted further investigation in the paediatric population and a Paediatric Investigation Plan was designed according to the requirements of the European Medicines Agency Paediatric Committee [15]. Only bilastine and rupatadine have been investigated in such a plan, among the second generation antihistamines. A phase III, double-blind, randomized, placebo-controlled, parallel-group clinical trial was carried out to assess the safety and tolerability of bilastine 10 mg once daily in children aged 2–11 years with allergic rhinoconjunctivitis or CU [28]. Several studies, aimed to determine the paediatric indication, were also conducted for cetirizine, levocetirizine, desloradine, fexofenadine and loratadine or rupatadine [29–34]; for pediatric use, local regulatory authorities still decide on the subject minimum age that can vary form 6 months to 12 years, for the same drug, in respect to the country.

To confirm the suitable dose in the paediatric population, a semi-mechanistic approach was applied to predict bilastine pharmacokinetic in children, assuming the same pharmacodynamic as described in adults. Performing dose-finding trials in children is not always ethical or feasible, particularly for younger ages. Importantly, it is in this latter group that dosing may be inadequate the most if maturation processes are not considered. The model was used to simulate the time evolution of plasma levels and wheal and flare effects after several doses. Simulations supported the selection of 10 mg/day in 2 to < 12 years old children [35, 36].

**Fig. 2:**
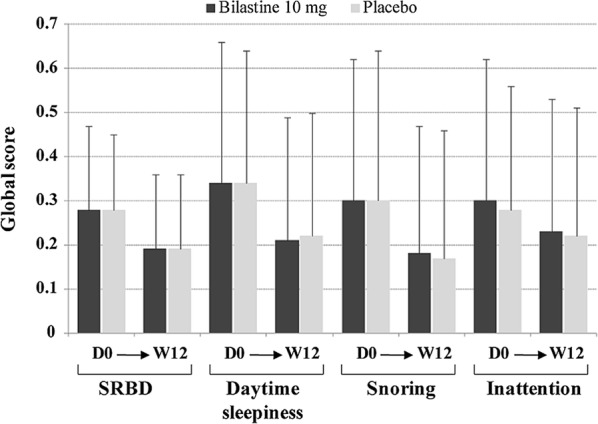
Assessment of somnolence/sedation from baseline (D0) to week 12 (W12) according to global scores on the four domains of the Pediatric Sleep Questionnaire: sleeping-related breathing disorder (SRBD), daytime sleepiness, snoring and inattention (reproduced with permission from 28)

The dose was chosen based on a previous modelling which was further confirmed by a paediatric pharmacokinetic study that established that a 10 mg dose of bilastine in children aged 2 to < 12 years provided an equivalent systemic exposure as a 20 mg dose in adults [35, 36]. Boys and girls aged 2–11 years, with a documented history of allergic rhinoconjunctivitis or CU and with clinical symptoms at study entry, were enrolled; after screening, 509 subjects were randomized. A bilastine 10 mg oral dispersible tablet (n = 260) or placebo (n = 249) was administered once daily in the morning under fasting conditions for 12 weeks. The primary analysis variable was the proportion of children in each treatment group without treatment-emergent adverse events (TEAEs) during the course of the study. Assessment of somnolence/sedation with the Pediatric Sleep Questionnaire (PSQ) was among the secondary variables. No statistically significant differences were found between treatment groups for incidences of TEAEs or related TEAEs in the population overall or by age subgroup. The majority of related TEAEs were mild to moderate in intensity. PSQ scores for somnolence/sedation decreased slightly from baseline to week 12 in both the bilastine 10 mg and placebo groups (Fig. 2). Between-group differences were not statistically significant for the total score or for scores in the individual domains [28].

#### Relevance of the non-sedating profile for children

Excessive daytime sleepiness (EDS) and associated learning, attention/hyperactivity, and conduct problems, in a general population sample of 1500 children were found to be mainly a manifestation of concomitant disease, including allergy, and not only a result of objective poor sleep [37]. Indeed, the symptoms of allergy can have detrimental effects on cognitive functions [38]. Treatment of urticaria, as recommended by current guidelines, aims at long-lasting control of symptoms and at well-being of the patient [1]. For children, this means that they should be helped to sleep comfortably and to avoid disease distress that may compromise school performance and conduct.

Urticaria treatment itself must not interfere with everyday life and school performance. Therefore, it is very important that CU be treated up to continuous control of symptoms and that a non-sedating antihistamine is used in this age group. First generation antihistamines have high sedating effects and also second-generation drugs may impact adversely on alertness and attention. Bilastine has a very low risk to induce somnolence, as suggested by pharmacological data, and demonstrated by clinical studies both in adults, and in children, that makes this drug a suitable treatment for children attending school [15, 20, 28, 39].

Sleepiness must not be induced by treatment as it has significant adverse effects on learning, mood and quality of life [40]. The relevance of this issue has been thoroughly investigated in a cohort of Italian primary school children. A significant worsening was detected in performance at complex task since mid-morning, concomitantly with sleepiness increase and significant correlations were found between subjective sleepiness and complex performance at all points [41]. As previously mentioned, Calhoun et al. [37] reported the association between EDS and impairment of parent reported learning, attention/hyperactivity, and conduct problems, in a general population sample of children, aged 6–12 years. Children underwent a 9-h polysomnogram, comprehensive neurocognitive testing, and parent rating scales. Results suggested that EDS impaired young school aged children’s ability to pay attention (e.g., concentration, listening, and distractibility) and level of activity (e.g., over-activity), and that this effect was large enough to be detected and reported by parents. Learning problems were reported by 57% of the parents whose children had EDS, suggesting that the sleepier they were, the higher the risk for difficulty in learning, incomplete and disorganized schoolwork, low grades, and trouble with reading, writing, and arithmetic. This was in agreement with previous reports [42].

In addition to learning and attention/hyperactivity problems, conduct problems (e.g., irritability and aggression) were associated with EDS [37].

Finally, both CU and its treatment could induce somnolence, and this effect would interfere with learning and cognitive activities. Based on data obtained in adults, bilastine has been authorised to treat children with CU for long periods to obtain control of symptoms. The excellent safety profile suggests that it could also have a favourable impact on school performance, cognitive activities and conduct in children.

## Conclusions

Chronic urticaria has a profoundly negative impact on quality of life and everyday life of affected children, impairing sleep and school and learning performance. Long-term, continuous treatment is required to control symptoms, so that great tolerability of a pharmacologic treatment is mandatory. First generation antihistamines cannot be considered safe for lack of evidence, and for their strong sedating effect; on this basis they are not recommended.

Bilastine is a suitable tool for treatment of CU, due to its efficacy and good tolerability profile that were proven in well-controlled studies using objective indices. Specifically, lack of potential to induce sedation allows prolonged administration without impairment of performance and learning abilities.

## Data Availability

Not applicable.

## Abbreviations

EAACI: European Academy of Allergology and Clinical Immunology
GA2LEN: Global Asthma and Allergy European Network
EDF: European Dermatology Forum
WAO: World Allergy Organization
CSU: chronic spontaneous urticaria
CIndU: chronic inducible urticaria
CU: chronic urticaria
PET: positron emission tomography
DLQI: Dermatology Life Quality Index
H_1_RO: H_1_ receptor occupancy
TEAE: treatment-emergent adverse events
EDS: excessive daytime sleepiness
